# Exposure to melamine exacerbates D‐galactose‐induced aging‐type toxicity in neuronal SH‐SY5Y cells

**DOI:** 10.1002/alz.088620

**Published:** 2025-01-03

**Authors:** Nitish Rai

**Affiliations:** ^1^ Department of Zoology, University of Lucknow, Lucknow India

## Abstract

**Background:**

Various investigations have elucidated the impact of diet and environmental toxins on the aging process. Melamine (Mel) is a widely recognized and infamous food adulterant with documented toxicity in various organs, including the brain. Nevertheless, there is currently a dearth of reports on the neurotoxic effects of Mel in aging neurons. This study aims to fill this knowledge gap by exploring the in‐vitro neurotoxicity induced by Mel in a D‐galactose (DG) induced aging model, utilizing neuronal SH‐SY5Y cells.

**Method:**

In this current investigation, the neuronal SH‐SY5Y cells underwent separate and combined treatments with D‐galactose (DG) and Melamine (Mel) to evaluate their neurotoxic potential. The assessment involved employing the MTT assay for cell viability and measuring neurite length. Additionally, we examined the activities of superoxide dismutase (SOD), catalase (CAT), and total antioxidant levels. Subsequent analyses included determining intracellular reactive oxygen species (ROS), mitochondrial membrane potential (MMP), and caspase‐3 (Casp3) activity.

**Result:**

When Melamine (Mel) and D‐galactose (DG) were co‐administered to neuronal SH‐SY5Y cells, a notable increase in cell death was observed compared to cells treated with DG or Mel individually, as well as untreated control cells. The co‐treated cells exhibited the most significant shrinkage in neurite length and the highest production of reactive oxygen species (ROS), indicating an exacerbated toxicity from Mel. Furthermore, the combined treatment (Mel + DG) led to a decrease in the activity of superoxide dismutase (SOD), catalase (CAT), and total antioxidants compared to cells treated with Mel or DG alone and untreated cells. Additionally, the co‐toxicity of Mel and DG significantly elevated the activity of caspase‐3 (Casp3) compared to all other groups, underscoring an enhanced apoptotic response.

**Conclusion:**

This study represents the initial exploration into the heightened neurotoxic potential of Melamine (Mel) within an aging model of neuronal SH‐SY5Y cells. These findings suggest that Mel consumption by the elderly could potentially contribute to elevated occurrences of neurodegenerative conditions such as Alzheimer’s disease and Parkinson’s disease.

